# Bilateral papillitis as the initial presentation of neurosyphilis in a patient previously treated for primary and secondary syphilis

**DOI:** 10.1016/j.ajoc.2022.101737

**Published:** 2022-10-31

**Authors:** Xiao Yi Zhou, Warren M. Sobol

**Affiliations:** Department of Ophthalmology, University Hospitals Eye Institute, Case Western Reserve University, Cleveland, OH, USA

**Keywords:** HIV, Papillitis, Syphilis, Uveitis

## Abstract

**Purpose:**

To report a patient previously treated for primary and secondary syphilis who presented with papillitis. The patient was found to have neurosyphilis likely due to inadequate treatment of primary and secondary syphilis.

**Observations:**

A 60-year-old male with human immunodeficiency virus (HIV) and hepatitis C was referred for evaluation of blurry vision for the past several months. Anterior segment examination was notable for 1+ diffuse non-granulomatous keratic precipitates and 2+ flare with trace cell in both eyes. Dilated fundus exam revealed grade 2 optic disc edema in both eyes with no evidence of infectious retinitis. He was recently treated for syphilis with a single dose of intramuscular (IM) penicillin. These findings were consistent with syphilitic papillitis likely secondary to neurosyphilis. The patient underwent a lumbar puncture which confirmed the diagnosis of neurosyphilis. He was admitted to the hospital for intravenous (IV) penicillin. He later revealed a prior history of syphilis that was treated 3 years ago and 1 year ago.

**Conclusions and importance:**

Ocular syphilis can have a wide variety of presentations. Any patient with syphilis and uveitis should have prompt work up for neurosyphilis. Patients with any stage of syphilis need close follow up with repeat titers after treatment to ensure adequate treatment and prevent progression and permanent ocular or neurologic sequelae.

## 1 Introduction

Cases of syphilis have been on the rise globally in recent years.^1^ There were over 133,000 cases of syphilis in the United States in 2020.^2^ Cases of primary and secondary syphilis in the United States has increased almost every year since 2000 and 2001.^2^ Most of these cases are attributable to increased cases among men, likely attributable to increases in cases among men who have sex with men.^2^^,^^3^

Ocular syphilis can be seen in all stages of syphilis, although eye manifestations most commonly occur in secondary and tertiary stages.^4^ Up to 70% of patients with ocular syphilis have reactive cerebrospinal fluid (CSF) Venereal Disease Research Laboratory (VDRL) test.^5^ We present a case of ocular syphilis in a patient with HIV and previously treated primary and secondary syphilis.

## 2 Case report

A 60-year-old male was referred for evaluation of bilateral blurry vision, eye pain, redness, photophobia and floaters, worse in the right eye, for the past several months. His ocular history was unremarkable. His medical history included HIV with non-compliance with antiviral medications for the past 4 months and recently diagnosed hepatitis C. The patient has a history of unprotected sex with men, and his last partner was over a year ago. He recently moved and established care with the infectious diseases service locally and had baseline laboratory tests 2 weeks prior to his ophthalmic examination that revealed elevated VDRL titers of 1:256. Of note, his VDRL was negative 5 years prior. Lab results also showed a CD4 count of 241 cells/mm^3^ and high HIV and hepatitis C viral load. He had no neurologic symptoms, gummatous lesions or cardiac signs or symptoms to suggest tertiary syphilis or neurosyphilis. His ocular symptoms were not considered to be related to syphilis at the time, thus he was treated with a single dose of IM penicillin and referred to ophthalmology for evaluation. He was restarted on anti-retroviral medications a week prior to the ophthalmic exam.

**Fig. 1:**
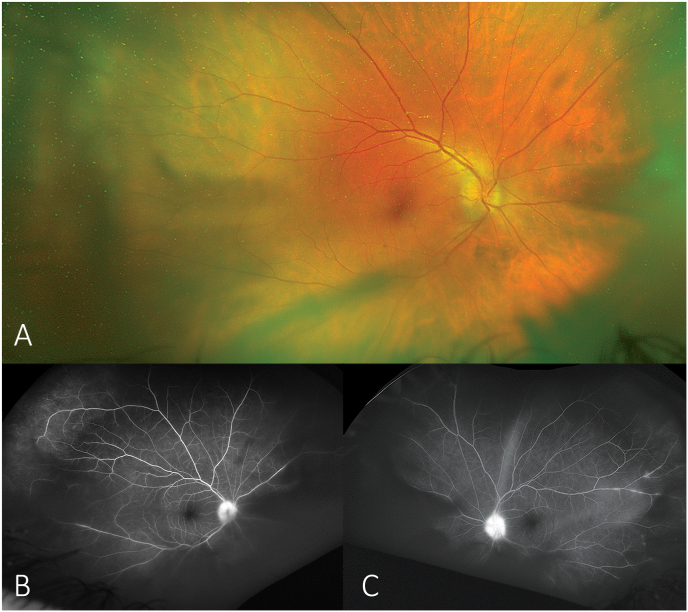
A, Fundus photograph of the right eye demonstrates optic nerve edema. B, Fluorescein angiogram of the right eye reveals hyperfluorescence of the disc and leakage from the central vein. C, Fluorescein angiogram of the left eye reveals similar findings of hyperfluorescence of the disc and leakage from the central vein.

On examination, his best corrected visual acuity (BCVA) was 20/50 in the right eye and 20/40 in the left eye. Anterior segment examination revealed 1+ diffuse non-granulomatous keratic precipitates and 2+ flare with trace cell in both eyes. Dilated fundus exam revealed grade 2 optic disc edema in both eyes with no evidence of infectious retinitis (Fig. 1A). Fluorescein angiography showed early and late hyperfluorescence of the discs in both eyes with low grade periphlebitis greater in the left eye than right eye (Fig. 1B and C). These findings were consistent with papillitis thought to be due to possible neurosyphilis. Patient was admitted to the hospital for further evaluation. Physical examination was remarkable for hyperreflexia of upper and lower extremities and a positive Babinski reflex on the right side. Computed tomography (CT) scan of the head was unremarkable. The patient underwent lumbar puncture which revealed normal opening pressure. The CSF was found to be reactive for VDRL, thus confirming the diagnosis of neurosyphilis, which requires positive CSF test, serology and neurologic signs or symptoms.^6^ He was treated with a 10-day course of IV penicillin.

The patient revealed during hospitalization that he had been treated for primary syphilis with IM penicillin 3 years prior to hospitalization and was treated again with IM penicillin 1 year prior to hospitalization when he developed a rash over his palms and soles thought to be due to secondary syphilis. He had no new symptoms or sexual contacts since he was last treated for syphilis.

One month after treatment, the patient's uncorrected visual acuity was 20/20 in the right eye and 20/60 in the left eye with improved optic disc edema and no evidence of active uveitis. Five months after treatment, VDRL titers decreased by four-fold, indicating adequate treatment. His BCVA a year after treatment was 20/20 in the right eye and 20/25 + 2 in the left eye.

## 3 Discussion

The differential diagnosis for anterior uveitis and papillitis is broad and includes sarcoidosis, leukemia, infectious etiologies such as toxoplasmosis, syphilis and tuberculosis. 7, 8, 9, 10, 11 Our patient's presentation was non-specific, however, in conjunction with his recent positive VDRL, the diagnosis of ocular syphilis should be highly considered and evaluated.***

Ocular syphilis is often called “the great masquerader” due to its variable and non-specific presentation and ability to affect any ocular structure. The most common presentation of ocular syphilis is panuveitis.^12^^,^^13^ However, in HIV-negative patients, the most common presentation is posterior uveitis.^14^ Both ocular and neurosyphilis can occur in any stage of syphilis.^15^ Anterior segment manifestations of ocular syphilis include chancre of the eyelid or conjunctiva, conjunctivitis, scleritis, keratitis, anterior uveitis, and hypopyon.^15^^,^^16^ Posterior segment manifestations include necrotizing or non-necrotizing retinitis, vitritis, vasculitis, chorioretinitis, perineuritis, optic neuritis and optic disc edema.^15^^,^^16^ There are two distinct clinical patterns that if present, can aid in diagnosis of ocular syphilis.^16^ The first is superficial retinal precipitates in syphilitic panuveitis, which consists of small superficial, creamy yellow precipitates that overlie areas of retinitis. These lesions resolve with minimal disruption of the retinal pigment epithelium. The second is acute syphilitic posterior placoid chorioretinitis (ASPPC), which is a large discrete yellowish circular area of outer retinal and inner choroidal inflammation involving the macula. As ocular syphilis can have many different presentations, a high index of suspicion is required for diagnosis.

Based on CDC guidelines at the time of the patient's diagnosis, any patient with syphilis and uveitis or other ocular manifestations should have a lumbar puncture with CSF examination to evaluate for neurosyphilis.^17^ The most recent CDC guidelines from 2021 recommend that patients with positive syphilis serology and ocular symptoms be promptly referred for a full ocular exam and cranial nerve evaluation.^6^ CSF examination is no longer necessary if ocular exam is abnormal, however CSF should be obtained if there is any cranial nerve dysfunction.^6^ CSF examination can be helpful in diagnosis of neurosyphilis in patient with ocular symptoms with a normal ocular and cranial nerve exam.^6^ Prompt diagnosis and treatment of ocular syphilis can prevent permanent vision loss.^18^ Ocular syphilis should be managed as neurosyphilis based on CDC guidelines, even when CSF is normal.^17^ Both ocular and neurosyphilis are treated with aqueous penicillin G 3–4 million units IV every 4 hours for 10–14 days.^6^ An alternative treatment option in reliable patients is a two-week course of daily intramuscular penicillin.^6^ As ocular syphilis and neurosyphilis was not initially suspected in our patient, the single dose of intramuscular penicillin that the patient received recently was not adequate treatment of ocular or neurosyphilis.

After treatment for ocular or neurosyphilis, close follow up of non-treponemal titers and possible repeat CSF analysis is important, especially in HIV patients, who are at higher risk for treatment failure or reinfection.^19^^,^^20^ Per the (Centers for Disease Control and Prevention) CDC guidelines, if serum titers normalize after treatment, then a repeat CSF evaluation is not necessary for patients without HIV or patients with HIV on effective anti-retroviral therapy.^6^ However, patients with persistent signs or symptoms of syphilis without evidence of reinfection or a sustained (>2 week) fourfold increase in non-treponemal titers are considered to have treatment failure.^6^ It is difficult to confirm without molecular testing whether a patient has syphilis reinfection or treatment failure.^21^ Our patient had a history of primary and secondary syphilis that was treated twice in the past three years with no evidence of reinfection, raising suspicion for treatment failure. Per the CDC guidelines, patients with treatment failure should have retreatment with weekly IM penicillin for 3 weeks unless there is evidence of neurosyphilis, which should be treated with IV penicillin.^6^ In this case, the patient's only symptom of syphilis were the ocular symptoms, which prompted further evaluation leading to the diagnosis of neurosyphilis.

In summary, ocular syphilis can have a wide variety of presentations. Any patient with syphilis and uveitis should have prompt ophthalmic and cranial nerve exam to evaluate for ocular and neurosyphilis. Patients with any stage of syphilis need close follow up with repeat titers after treatment to ensure adequate treatment and prevent progression and permanent ocular or neurologic sequelae.

## Patient consent

Consent to publish the case report was not obtained. This report does not contain any personal information that could lead to the identification of the patient.

## Funding

No funding or grant support.

## Declaration of competing interest

None.
